# SNiPER: a novel hypermethylation biomarker panel for liquid biopsy based early breast cancer detection

**DOI:** 10.18632/oncotarget.27303

**Published:** 2019-11-05

**Authors:** Jolein Mijnes, Janina Tiedemann, Julian Eschenbruch, Janina Gasthaus, Sarah Bringezu, Dirk Bauerschlag, Nicolai Maass, Norbert Arnold, Jörg Weimer, Tobias Anzeneder, Peter A. Fasching, Matthias Rübner, Benjamin Bruno, Uwe Heindrichs, Jennifer Freres, Hanna Schulz, Ralf-Dieter Hilgers, Nadina Ortiz-Brüchle, Sonja von Serenyi, Ruth Knüchel, Vera Kloten, Edgar Dahl

**Affiliations:** ^1^Molecular Oncology Group, Institute of Pathology, University Hospital RWTH Aachen, Aachen, Germany; ^2^RWTH centralized Biomaterial Bank (RWTH cBMB) at the Institute of Pathology, University Hospital RWTH Aachen, Aachen, Germany; ^3^Department of Gynecology and Obstetrics, University Medical Centre Schleswig-Holstein, Campus Kiel, Kiel, Germany; ^4^Patients' Tumor Bank of Hope (PATH-Biobank) Foundation, München, Germany; ^5^Department of Gynecology and Obstetrics, University Hospital Erlangen, Erlangen, Germany; ^6^Institute of Clinical Molecular Biology, University Medical Centre Schleswig-Holstein, Campus Kiel, Christian-Albrechts-University, Kiel, Germany; ^7^Department of Gynecology and Obstetrics Luisenhospital, Aachen, Germany; ^8^Institute of Medical Statistics, University Hospital RWTH Aachen, Aachen, Germany; ^9^Current address: Bayer AG, Pharmaceuticals Division, Biomarker Research, Wuppertal, Germany; ^*^Share equal senior authorship

**Keywords:** liquid biopsy, breast cancer, early detection, hypermethylation, discriminative CpG dinucleotides

## Abstract

**Introduction:**

Mammography is the gold standard for early breast cancer detection, but shows important limitations. Blood-based approaches on basis of cell-free DNA (cfDNA) provide minimally invasive screening tools to characterize epigenetic alterations of tumor suppressor genes and could serve as a liquid biopsy, complementing mammography.

**Methods:**

Potential biomarkers were identified from The Cancer Genome Atlas (TCGA), using HumanMethylation450-BeadChip data. Promoter methylation status was evaluated quantitatively by pyrosequencing in a serum test cohort (*n =* 103), a serum validation cohort (*n =* 368) and a plasma cohort (*n =* 125).

**Results:**

*SPAG6*, *NKX2-6* and *PER1* were identified as novel biomarker candidates. *ITIH5* was included on basis of our previous work. In the serum test cohort, a panel of *SPAG6* and *ITIH5* showed 63% sensitivity for DCIS and 51% sensitivity for early invasive tumor (pT1, pN0) detection at 80% specificity. The serum validation cohort revealed 50% sensitivity for DCIS detection on basis of *NKX2-6* and *ITIH5*. Furthermore, an inverse correlation between methylation frequency and cfDNA concentration was uncovered. Therefore, markers were tested in a plasma cohort, achieving a 64% sensitivity for breast cancer detection using *SPAG6*, *PER1* and *ITIH5*.

**Conclusions:**

Although liquid biopsy remains challenging, a combination of *SPAG6*, *NKX2-6*, *ITIH5* and *PER1* (SNiPER) provides a promising tool for blood-based breast cancer detection.

## Introduction

Breast cancer remains the most frequently diagnosed cancer and second leading cause of cancer deaths amongst women worldwide [1]. Early localized and ductal carcinoma *in situ* (DCIS) show an excellent 5-year survival of nearly 100%, this rate however decreases to only 27% in metastatic breast cancer [2]. Despite recent advances in the clinical treatment of breast cancer, detection of the disease in an early stage remains key to successful outcome [3].

The current gold standard for early breast cancer detection is mammography [4]. Mammography is able to detect small invasive breast tumors before they become palpable and is the most effective tool for detection of micro calcifications and DCIS [5]. Nevertheless, the use of mammography remains controversial. Mammography causes personal discomfort, resulting in insufficient compliance rates [6, 7]. Moreover, it has poor accuracy in women with dense breast tissue, causing a decrease in sensitivity from 70–91% to 30–48% [5, 6, 8–10], and is less sensitive for the detection of small or diffuse tumors [11]. Additionally, due to similar appearance of malignant and benign breast lesions many unnecessary biopsies are taken [5, 8, 12]. Conventional blood-based cancer tests, relying on the detection of serum markers CA15.3 and carcinoma embryonic antigen (CEA), are ineffective as they are not breast cancer specific and only 10% of early breast cancers show increases [3]. Therefore, we are in need of a minimally invasive tool to increase compliance and improve non-invasive screening.

Non-invasive methods based on the analysis of circulating cell-free DNA (cfDNA) in bodily fluids provide opportunities for new diagnostic approaches [13]. In healthy individuals, the majority of cfDNA in blood is derived from hematopoietic cells. In cancer patients, increased levels of cfDNA are observed, of which < 0.1% to > 10% [14] is tumor-derived and termed circulating tumor DNA (ctDNA) [15]. Primary and metastatic breast tumors shed significant amounts of ctDNA into the bloodstream mainly through cellular apoptosis and necrosis [16]. The quantity of ctDNA correlates to tumor stage, as ctDNA is detectable in 90% of all stage breast tumors, whereas only 50% of patients with stage I breast cancer show detectable ctDNA levels [17]. Tumor cell spread may however already occur in DCIS [18–20]. Due to the origin of ctDNA, the genetic and epigenetic alterations found in ctDNA reflect the genome and epigenome of the cell of origin [13]. Besides being a frequently observed phenomenon, epigenetic changes, like CpG hypermethylation, are a very early event in carcinogenesis [4, 21], making it an excellent tool for early breast cancer detection.

Although the diagnostic potential of methylation-based biomarkers in breast cancer has been recognized and investigated, none of the proposed markers have reached clinical application, mainly due to limitations in study design. So far, most studies have not considered promoter methylation of identified genes in large (> 200 samples) or more importantly, independent sets of samples [22–25]. Notably, there was a lack of distinct specificity controls, such as age-matched healthy or benign disease controls [23–26]. Moreover, studies included patients with breast tumors ranging from pT1 to pT4, making it difficult to determine the value of biomarkers for early breast cancer detection [23–28]. Of additional importance is the lack of statistics, which take in account the influence of age on methylation levels [23, 25, 26].

To address these limitations, in the present study we considered: (1) promoter methylation of biomarker genes in 363 samples of breast cancer patients and 233 age-matched benign controls, (2) only patients with non-invasive DCIS (pTis) and small localized tumors (pT1) without lymph node (pN0) and distant metastasis (pM0), and (3) a systematic statistical workflow for quantitative methylation analysis. Accordingly, we identified *SPAG6*, *PER1* and *NKX2-6* as novel potential biomarkers for minimally invasive breast cancer detection.

## Results

### Novel breast cancer biomarker candidates *SPAG6*, *PER1* and *NKX2-6* identified using TCGA

**Figure 1:**
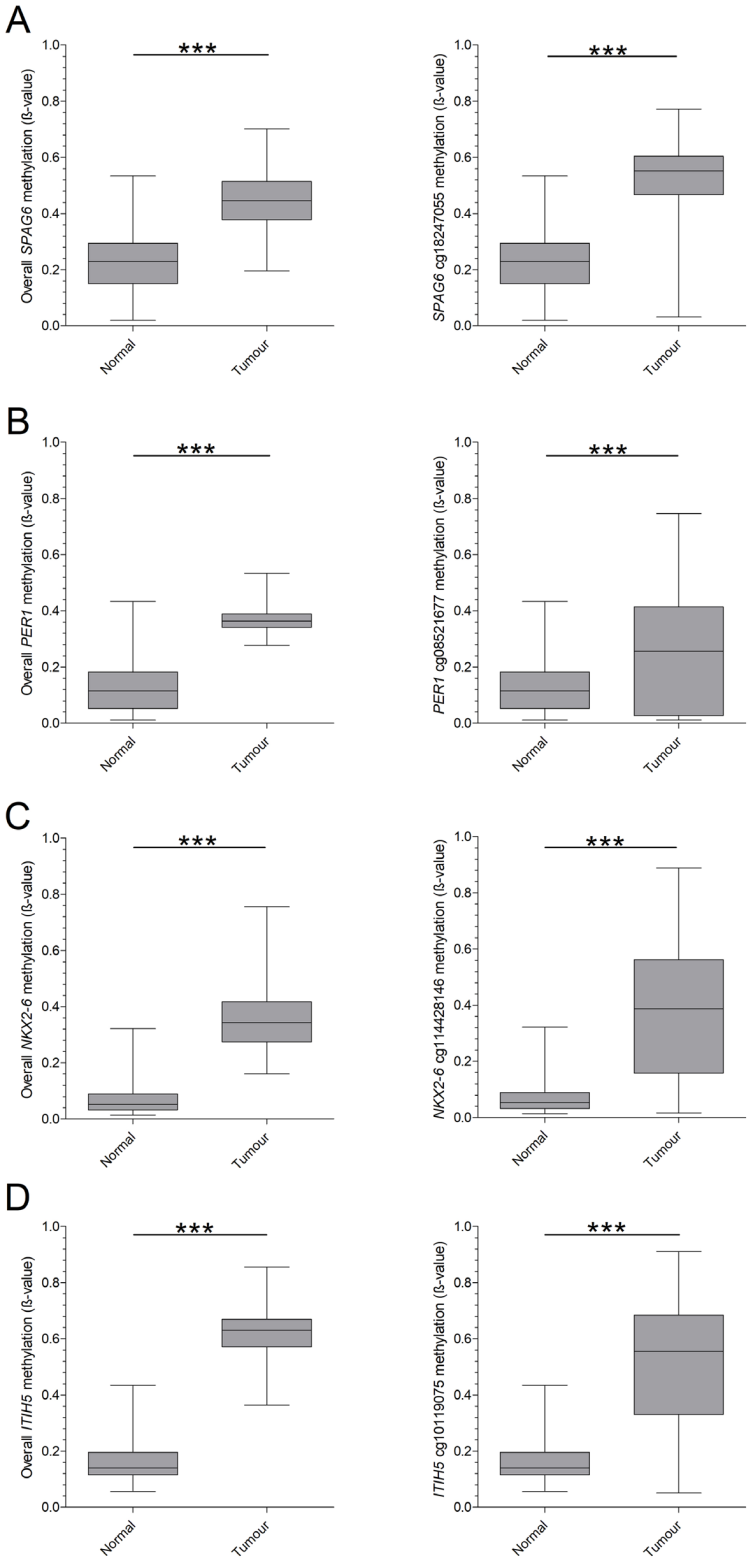
TCGA based overall- and CpG-specific methylation of candidate biomarkers in pT1 breast cancer. Biomarkers were identified on basis of the TCGA database, by plotting the overall methylation pattern of healthy breast tissue and breast cancer tissue (pT1 only). In addition, single CpGs were plotted to determine a specific region of interest. (**A**) *SPAG6* showed a significant increase in mean methylation in breast cancer and CpG cg18247055. (**B**) For *PER1* a significant difference was found as well, for both mean methylation and CpG cg08521677. (**C**) *NKX2-6* presented an increase in methylation in mean methylation and for CpG cg14428146, which was significant in both cases. (**D**) Overall methylation frequency and CpG cg10119075 for *ITIH5* was significantly higher in breast cancer. ^*^ *p* < 0.05, ^**^ *p* < 0.01, ^***^ *p* < 0.001, ns: non-significant. Whiskers indicate minimum, 25% percentile, median, 75% percentile and maximum.

Based on TCGA analysis and the defined criteria, we identified ten potential candidate genes of which *SPAG6*, *PER1* and *NKX2-6* proved suitable for early breast cancer detection after an initial validation in breast cancer cell lines and a small cryoconserved tissue cohort (Supplementary Figure 1). *ITIH5* was included on basis of previous promising data by our group [29]. A significant increase in overall methylation level in breast cancer patients with pT1 tumors, compared to healthy subjects was seen for mentioned genes (*p* < 0.0001, Figure 1A–1D, left panel). In more detail, we sought for specific CpGs in promoter regions, which where cg18247055 (*SPAG6*), cg08521677 (*PER1*), cg14428146 (*NKX2-6*) and cg10119075 (*ITIH5*, Figure 1A–1D, right panel). Using these promoter specific CpGs stronger differences in methylation frequency between healthy subjects and breast cancer patients were found, as indicated by higher fold change (FC). An overall FC of 1.33 was found for *SPAG6*, which was increased to 1.72 when including only cg18247055. *PER1* cg08521677 showed a FC of 1.64 whereas the overall FC was 1.11. A high increase in FC was seen for *NKX2-6* cg14428146 (3.58) and *ITIH5* cg10119075 (2.30) compared to overall FC (1.37 and 0.98, respectively). Basal-like breast cancers frequently show low methylation levels of tumor suppressor genes, single CpGs of *SPAG6*, *PER1*, *NKX2-6* and *ITIH5* however showed a higher methylation frequency in this molecular breast cancer subtype (59%, 42%, 52% and 41%, respectively).

### Technical sensitivity and specificity of pyrosequencing assays

CpGs showing a high FC in the promoter regions of the candidates were used to guide assay design. For *SPAG6* in total ten CpG sites were included in the pyrosequencing assay, whereas for *PER1* two sites were investigated. Assays for *NKX2-6* and *ITIH5* both covered four CpGs (Supplementary Figures 2–5). Before starting methylation analysis in patient samples, technical sensitivity and specificity of the assays was evaluated. A dilution series with increasing amounts of fragmented unmethylated- and decreasing amounts of fragmented *in vitro* methylated lymphocyte bisulfite DNA was implemented to test technical specificity. The observed methylation values were plotted against the expected methylation frequency and linear regression analysis was performed (Supplementary Figure 6). Pyrosequencing assays for *NKX2-6* and *ITIH5* demonstrated an excellent performance, with correlation coefficients of 0.98 (Supplementary Figure 6C–6D). Assays for *SPAG6* and *PER1* showed a correlation coefficient of 0.88 and 0.89, respectively (Supplementary Figure 6A–6B). In addition, the lower detection limit e.g. technical sensitivity, of each assay was tested. For this purpose, decreasing amounts of fragmented *in vitro* methylated lymphocyte DNA were spiked into 1 ml of pooled serum or plasma (three, respectively, four healthy donors) before DNA isolation. The obtained methylation frequencies were plotted and a line was fitted (Supplementary Figure 7). Lines for *SPAG6* (serum R^2^: 0.79, plasma R^2^: 0.85), *PER1* (serum R^2^: 0.91, plasma R^2^: 0.87), *NKX2-6* (serum R^2^: 0.98, plasma R^2^: 0.97) and *ITIH5* (serum R^2^: 0.54, plasma R^2^: 0.86) showed good correlations. The limits of detection for the different pyrosequencing assays were 2.77 ng (*SPAG6*), 1.64 ng (*PER1*), 0.64 ng (*NKX2-6*) and 4.75 ng (*ITIH5*) in serum. In plasma the limit of detection was lower for *SPAG6* and *ITIH5* (1.39 ng and 2.03 ng, respectively) and in the same range for *PER1* and *NKX2-6* (1.95 ng and 0.97 ng, respectively).

### High sensitivity for DCIS- and early invasive breast cancer detection in test cohort

**Figure 2:**
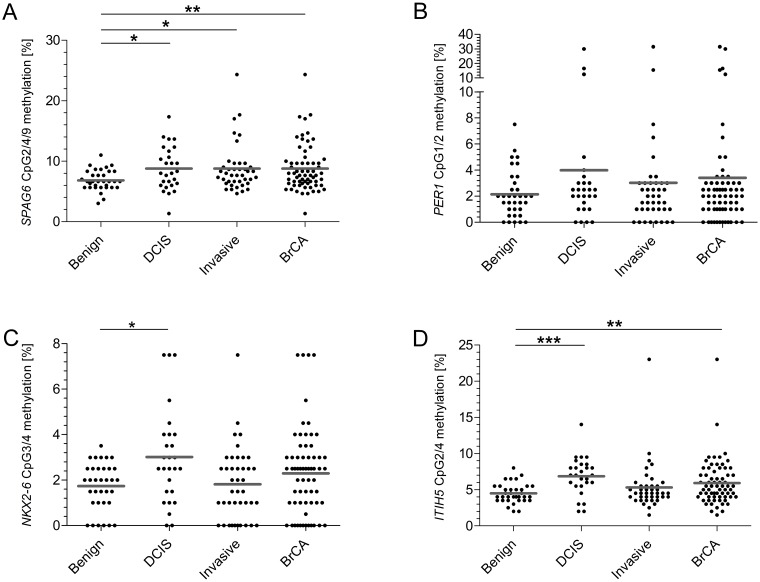
On basis of FC determined CpGs, *SPAG6*, *NKX2-6* and *ITIH5* show significantly increased methylation frequencies in the test cohort. (**A**) For *SPAG6* CpG 2, 4 and 9 showed the best discrimination. Comparing benign controls to cases significant differences were found (DCIS *p* = 0.0258, invasive breast cancer *p* = 0.0168, overall breast cancer *p* = 0.0073). (**B**) The best CpG for *PER1* were 1 and 2, no statistically significant different differences were found between the groups (DCIS *p* = 0.2758, invasive breast cancer *p* = 0.9359, overall breast cancer *p* = 0.5554). (**C**) CpG 3 and 4 showed most discriminative for *NKX2-6*, the methylation frequency in DCIS cases showed significantly higher (DCIS *p* = 0.0201, invasive breast cancer *p* = 0.8928, overall breast cancer *p* = 0.2443). (**D**) For *ITIH5* CpG 2 and 4 were identified as most discriminative showing significant differences comparing benign controls to DCIS patients and all breast cancer patients (DCIS *p* < 0.0001, invasive breast cancer *p* = 0.3079, overall breast cancer *p* = 0.0085). ^*^ *p* < 0.05, ^**^ *p* < 0.01, ^***^ *p* < 0.001, ns: non-significant. Grey line indicates mean methylation level.

We initially assessed promoter methylation of *SPAG6*, *PER1*, *NKX2-6* and *ITIH5* in a serum cohort consisting of samples of women with benign disease (*n* = 34), DCIS (*n* = 27) and early invasive breast cancer (*n* = 42). The CpGs in the regions of interest showed a rather heterogeneous methylation pattern, with a mean methylation level varying from 2.9% to 13.2% for breast cancer cases (Supplementary Figure 8). We therefore decided to work with a combination of CpGs showing the highest discrimination instead of mean methylation levels per gene, as supported by TCGA analysis of single CpGs. To determine which CpGs displayed the highest methylation frequencies in breast cancer patients compared to benign controls (discriminative CpGs), different statistical strategies were used; FC and generalized linear model (GLM) with co-factor age [30], *t*-test and ROC analysis on single CpGs to strengthen results. Applying the FC method CpG2/4/9 in the *SPAG6* assay showed the highest discrimination between cases and controls. For *PER1* CpG1/2 showed the highest FC, for *NKX2-6* CpG3/4, and for *ITIH5* CpG2/4. Using these discriminative CpGs on basis of FC, *SPAG6* showed a significant higher methylation level in breast cancer (mean of 6.84% in benign controls versus 8.79% in breast cancer, *p* = 0.0073), DCIS (6.84% versus 8.79%, *p* = 0.0258) and early invasive breast cancer (6.84% versus 8.80%, *p* = 0.0168, Figure 2A). *ITIH5* showed significant increases in methylation level for breast cancer patients (4.49% versus 5.93%, *p* = 0.0085) and DCIS patients (4.49% versus 6.87%, *p* < 0.0001, Figure 2D). *NKX2-6* showed a significant higher methylation in DCIS patients (1.74% versus 3.02%, *p* = 0.0201, Figure 2C), whereas *PER1* showed no significant differences (Figure 2B). ROC analysis was then performed, using only discriminative CpGs, to evaluate sensitivity and specificity of single biomarkers and biomarker combinations for breast cancer detection (Table 1). *SPAG6* shows, at a cut-off methylation of 8.5% and specificity of 82.3%, an equal sensitivity for DCIS- (44%) and early invasive breast cancer (39%) detection. Whereas *ITIH5* shows a high sensitivity for DCIS (74%) detection, sensitivity for early invasive breast cancer detection is strongly decreased (22%, cut-off of 5.8%, 85.3% specificity). A combination of *SPAG6* and *ITIH5* shows the best performance, with 63% sensitivity for DCIS- and 51% sensitivity for early invasive cancer detection (cut-off 6.7% and 79.4% specificity). Adding *PER1* or *NKX2-6* to the two-gene panel increases sensitivity for DCIS detection to 70%, although decreases sensitivity for early invasive breast cancer (39% and 41%, respectively) detection. The same holds true for a four-gene panel (Table 1). On basis of the more stringent GLM, significantly higher methylated CpGs were confirmed for *SPAG6* and *ITIH5*.

**Table 1:** Sensitivity and specificity of single markers and biomarker combinations in the serum test cohort

		**AUC**	**Significance**	**Sensitivity (%)**	**Specificity (%)**	**Cut-off (%)**
***SPAG6***	DCIS	0.6672	0.0259	44	82	8.5
	Invasive	0.6610	0.0169	39	82	
	BC	0.6635	0.0073	41	82	
***ITIH5***	DCIS	0.7985	< 0.0001	74	85	5.8
	Invasive	0.5685	0.3095	22	85	
	BC	0.6598	0.0087	43	85	
***SPAG6 - PER1***	DCIS	0.7146	0.0042	48	79	6.3
	Invasive	0.6395	0.0385	32	79	
	BC	0.6693	0.0055	38	79	
***SPAG6 - NKX2-6***	DCIS	0.7249	0.0027	59	79	5.7
	Invasive	0.6438	0.0329	41	79	
	BC	0.6766	0.0039	49	79	
***SPAG6 - ITIH5***	DCIS	0.7985	< 0.0001	63	79	6.7
	Invasive	0.6567	0.0201	51	79	
	BC	0.7130	0.0005	51	79	
***SPAG6 - PER1 - NKX2-6***	DCIS	0.7424	0.0012	56	79	4.6
	Invasive	0.6291	0.0555	44	79	
	BC	0.6734	0.0044	49	79	
***SPAG6 - ITIH5 - NKX2-6***	DCIS	0.8404	< 0.0001	70	79	5.5
	Invasive	0.6697	0.0119	41	79	
	BC	0.7379	< 0.0001	53	79	
***SPAG6 - PER1 - ITIH5***	DCIS	0.8061	< 0.0001	70	79	5.6
	Invasive	0.6392	0.0390	39	79	
	BC	0.7063	0.0007	51	79	
***SPAG6 - PER1 - ITIH5 - NKX2-6***	DCIS	0.8415	< 0.0001	70	79	4.7
	Invasive	0.6481	0.0280	39	79	
	BC	0.7184	0.0003	51	79	

Note: The following CpGs were used for ROC analysis: *SPAG6* CpG 2, 4 and 9, *PER1* CpG 1 and 2, *NKX2-6* CpG 3 and 4, *ITIH5* CpG 2 and 4. Only significant results are shown. AUC: Area under the curve, BC: breast cancer.

### *SPAG6, PER1, NKX2-6* and *ITIH5* validation in an independent cohort

To further evaluate biomarker performance and to validate initial results, the candidates were tested in an independent serum cohort, consisting of patients with benign disease (*n* = 185), DCIS (*n* = 26) and early invasive breast cancer (*n* = 157). The CpGs that were selected on basis of FC and GLM in the test cohort were tested in the samples of the validation cohort as well. Employing the previously selected CpGs, *PER1* showed a significant higher methylation level in breast cancer patients (mean of 2.58% in benign controls versus 2.87% in breast cancer cases, *p* = 0.0172) and early invasive breast cancer patients (2.6% versus 2.98%, *p* = 0.0058). DCIS patients showed a significant decrease in *NKX2-6* methylation (2.64% versus 1.64%, *p* = 0.0084) compared to benign controls. *SPAG6* and *ITIH5* did not show significant increases in methylation frequency. The CpGs selected in the test cohort, worked particularly well for the detection of DCIS in the validation cohort; *NKX2-6* alone showed a sensitivity of 42% (cut-off methylation 1.3%, 79% specificity) for DCIS detection, which increased to 50% by adding *ITIH5* (cut-off 2.9%, 77% specificity). A four gene combination performed equally well for DCIS detection as *NKX2-6* alone (Supplementary Table 1). A separate FC and GLM analysis was performed for the validation cohort as well; the most discriminative CpGs differed from those in the test cohort for *SPAG6*, *NKX2-6* and *ITIH5*. Applying a FC, CpG3/4/8 in *SPAG6*, CpG1/2/4 in *NKX2-6* and CpG2/3 in *ITIH5* demonstrated discriminative. Subsequent ROC curve analysis on basis of validation cohort specific CpGs revealed significant results for a combination of *SPAG6* and *PER1*, achieving breast cancer detection with 25% sensitivity (Supplementary Table 2). On basis of GLM, CpG 3 in *NKX2-6* revealed the highest discriminative power and presented significant differences comparing the methylation levels of DCIS (2.6% versus 1.6%, *p* = 0.0030), invasive breast cancer (2.6% versus 2.2%, *p* = 0.0070) and overall breast cancer (2.6% versus 2.1%, *p* = 0.00110) to benign controls. ROC curve analysis for *NKX2-6* CpG3 revealed a sensitivity of 38% for DCIS detection, which was decreased to 25% for breast cancer detection, both at 84% specificity (Supplementary Table 2).

### Methylation frequency does not differ across different locations

**Figure 3:**
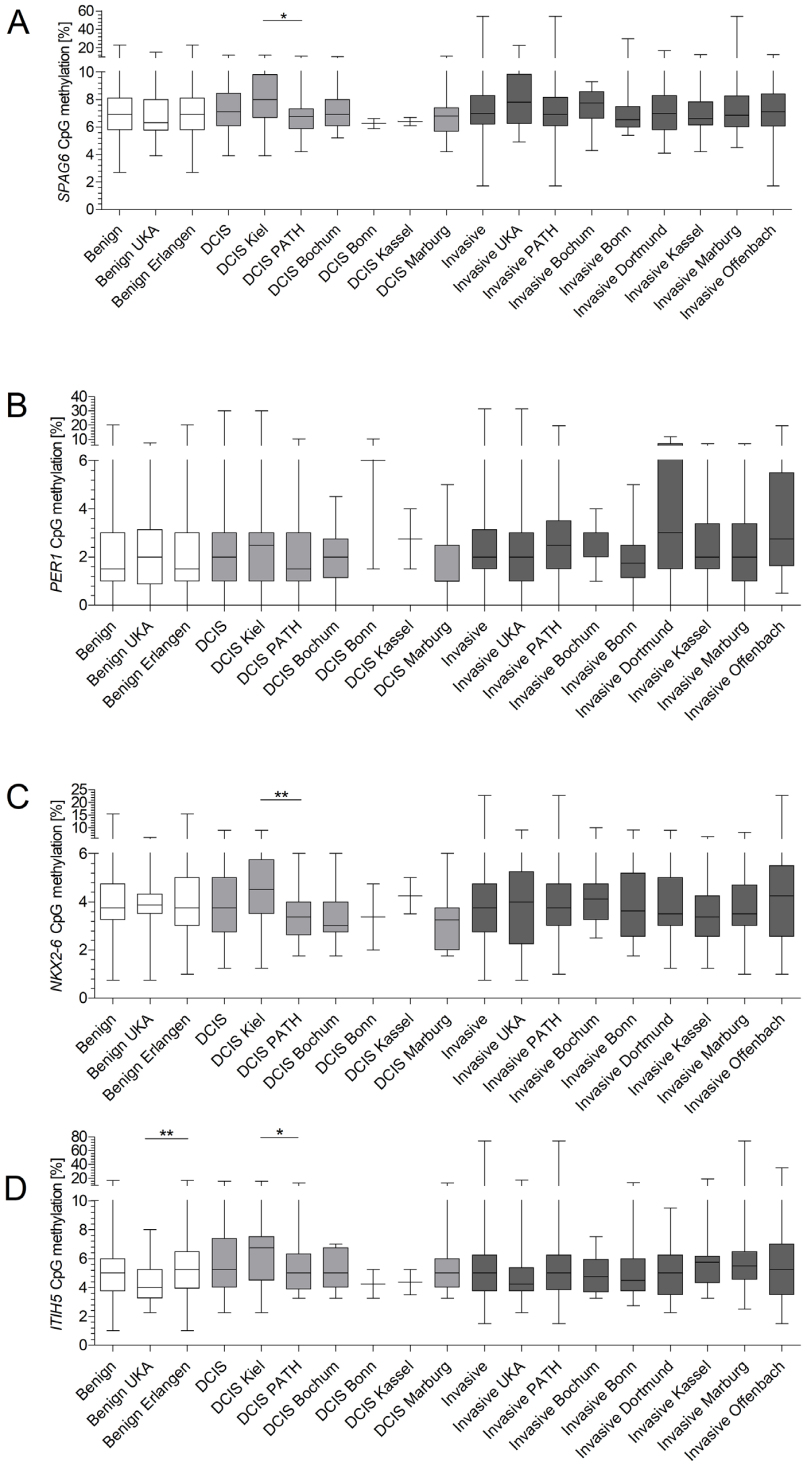
Site-specific methylation frequencies of *SPAG6*, *PER1*, *NKX2-6* and *ITIH5* (**A**–**D**). Methylation levels of benign diseased, DCIS patients and invasive breast cancer patients were compared across all sites of which serum was obtained. *ITIH5* showed a significant difference in methylation levels for benign samples of the test- and validation cohort (*p* = 0.0038). DCIS samples from the test cohort showed a significant higher methylation frequency for *SPAG6* (*p* = 0.0133), *NKX2-6* (*p* = 0.0064) and *ITIH5* (*p* = 0.0259) compared to the validation cohort. Whiskers indicate minimum, 25% percentile, median, 75% percentile and maximum.

As we could not confirm our initial promising results in an independent patient cohort, we sought for reasons for this discrepancy. The sera from the test cohort were derived from RWTH cBMB and UKSH, whereas the validation cohort consisted of breast cancer sera from PATH-Biobank, which receives material from multiple certified breast cancer centers in Germany (Bochum, Bonn, Dortmund, Kassel, Marburg and Offenbach) and benign samples of university hospital Erlangen. We speculated that methylation frequencies might vary depending on hospital of sample collection and therefore compared methylation levels across the different hospitals. Benign samples did not show any significant differences in methylation level for *SPAG6*, *PER1* and *NKX2-6* (Figure 3A–3C). Methylation frequency of *ITIH5* was however significantly higher (*p* = 0.0038) in benign samples of the validation cohort (Figure 3D). Comparing methylation levels of DCIS samples from all sites revealed no significant differences. However, significant higher methylation frequencies for *SPAG6*, *NKX2-6* and *ITIH5* were observed in DCIS samples from the test- compared to the validation cohort (Figure 3A–3D). Methylation levels of patients with early invasive breast cancer showed no significant differences in methylation (Figure 3A–3D).

### cfDNA concentration inversely correlates to methylation level

**Figure 4:**
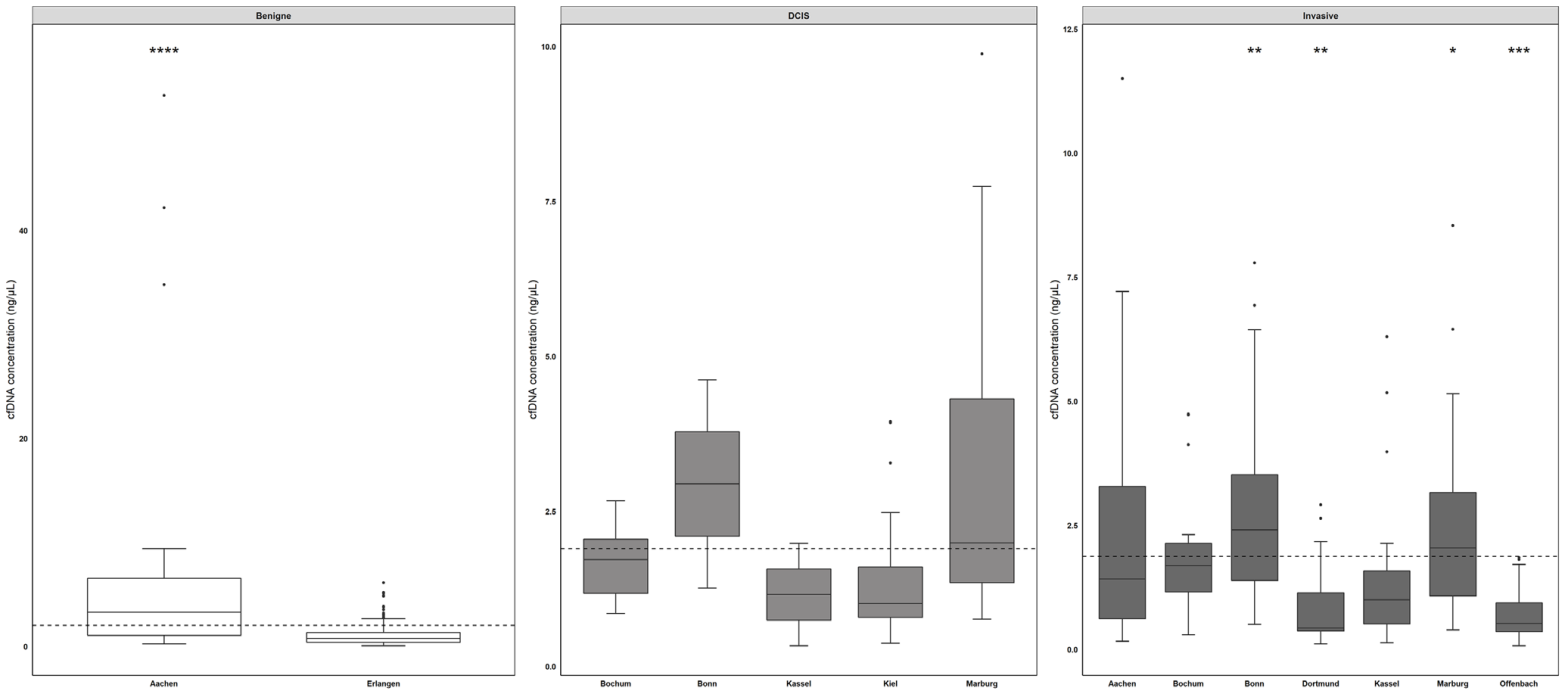
cfDNA concentrations across different sites differ significantly. Comparing cfDNA levels across all sites, benign samples derived from RWTH cBMB (mean concentration 7.0 ng/µl, range) showed the highest cfDNA concentrations, followed by samples from Bonn (DCIS samples mean concentration of 2.94 ng/µl, invasive breast cancer samples mean concentration 2.90 ng/µl) and Marburg (DCIS samples mean concentration 3.34 ng/µl, invasive breast cancer samples mean concentration 2.37 ng/µl). Benign samples derived from Aachen showed a significantly increased cfDNA concentration compared to samples from Erlangen and invasive breast cancer samples from Dortmund and Offenbach. DCIS samples from Marburg showed the highest cfDNA concentration in DCIS samples. Samples from invasive breast cancer patients obtained from Bochum and Marburg showed significant increased cfDNA concentrations compared to Offenbach and Dortmund. ^*^ *p* < 0.05, ^**^ *p* < 0.01, ^***^ *p* < 0.001. Whiskers indicate minimum, 25% percentile, median, 75% percentile and maximum.

**Figure 5:**
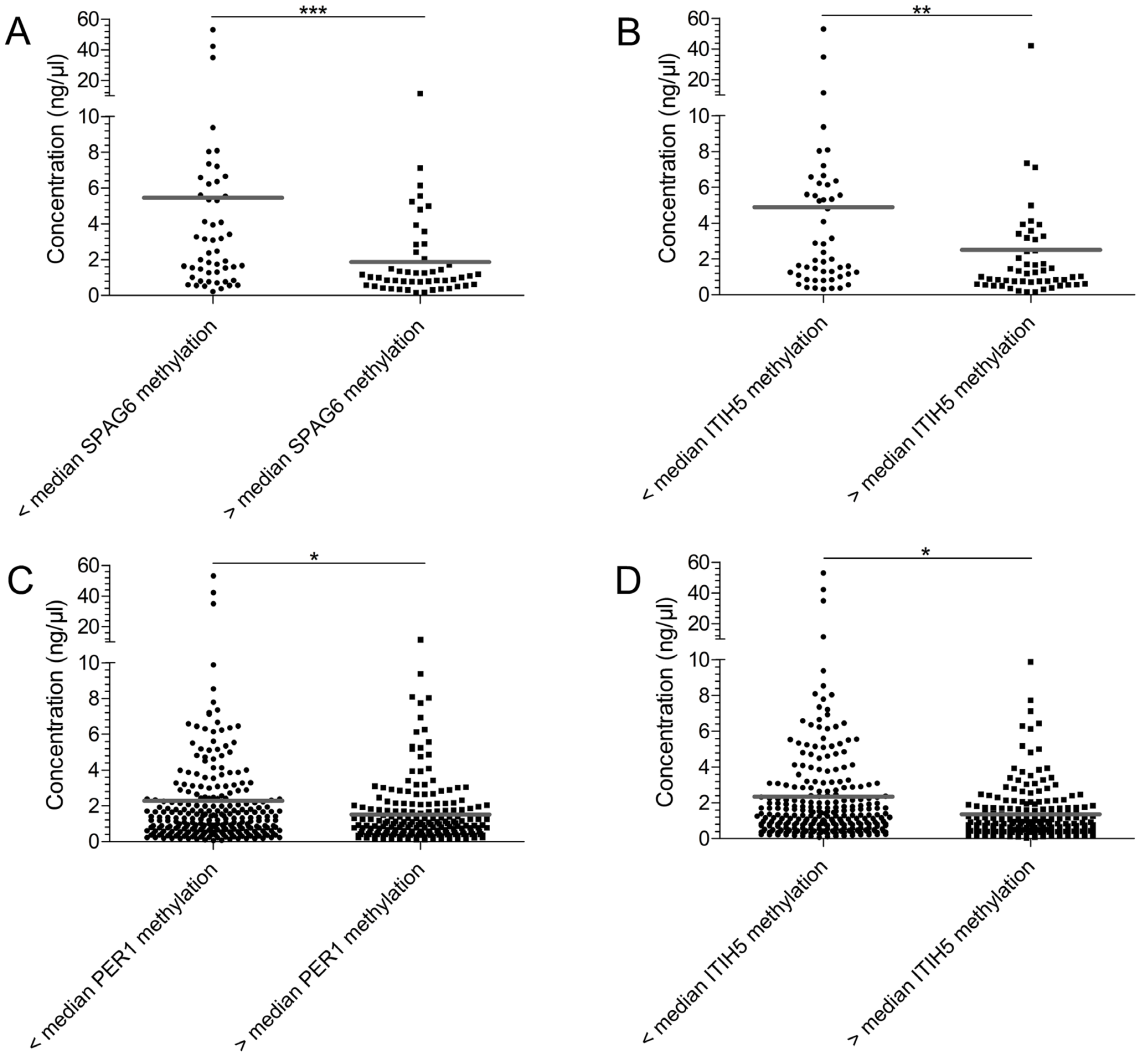
Samples with a high median methylation level show a decreased cfDNA concentration. The cfDNA concentration of samples was plotted according to methylation level, either below or above median methylation level: test cohort *SPAG6* 7.25% (**A**) and *ITIH5* 4.88% (**B**), validation cohort *PER1* 2.0% (**C**) and *ITIH5* 5.25% (**D**). In the test cohort samples showing a below median methylation level, had a significantly higher methylation level for *SPAG6* (*p* = 0.0006) and *ITIH5* (*p* = 0.0024). In the validation cohort the same was observed for *ITIH5* (*p* = 0.0013) and *PER1* (*p* = 0.0118). ^*^ *p* < 0.05, ^**^ *p* < 0.01, ^***^ *p* < 0.001, ns: non-significant. Grey line indicates mean methylation level.

In addition to comparing methylation levels across sites, we investigated a possible correlation between methylation frequency and cfDNA concentration. Kruskal-Wallis analysis revealed that benign samples from RWTH cBMB showed the highest cfDNA concentrations, followed by samples from Bonn and Marburg (Figure 4). An inverse relationship between cfDNA concentration and methylation level was found when plotting cfDNA concentration into groups: below median methylation- and above median methylation level (Figure 5). In the test cohort, *SPAG6* and *ITIH5* showed a significant difference in cfDNA concentration, with the highest cfDNA concentrations in the below median methylation group (*p* = 0.0006 and *p* = 0.0024, respectively, Figure 5A–5B). In the validation cohort the same was shown for *PER1* and *ITIH5* (*p* = 0.0013 and *p* = 0.0118, respectively, Figure 5C–5D). Spearman correlation analysis confirmed an inverse relationship between cfDNA concentration and methylation level, with correlation coefficients of -0.3647 (*p* = 0.0002) and -0.3009 (*p* = 0.0022) for *SPAG6* and *ITIH5* in the test cohort. Correlation coefficients of -0.1336 for *PER1* (*p* = 0.0040) and -0.1155 for *ITIH5* (*p* = 0.0130) were found in the validation cohort. Therefore, ROC curve analysis including only samples with a cfDNA concentration below median was performed in the validation cohort. *PER1* showed an 11% increase in sensitivity for invasive breast cancer detection (cut-off methylation 3.8%, 31% sensitivity, 82% specificity), compared to analysis of all samples. Using CpG2/4 for *NKX2-6* sensitivity for DCIS was increased with 18% (cut-off 0.8%, 60% sensitivity, 89% specificity). In case *NKX2-6* CpG3 was included, DCIS could be detected with 60% sensitivity, which is an 22% increase (cut-off 1.5%, 82% specificity). ROC analysis on CpG2/4 in *ITIH5* resulted in an 29% increase in sensitivity for DCIS detection (cut-off 3.8%, 60% sensitivity, 79% specificity).

### Highest sensitivity and specificity for breast cancer detection in a plasma cohort

Since plasma shows lower cfDNA concentrations compared to serum, we tested the methylation frequency of *SPAG6*, *PER1*, *NKX2-6* and *ITIH5* in a plasma cohort, consisting of women with a benign breast disease (*n* = 14) and invasive breast cancer (*n* = 111). Compared to serum cfDNA levels, the cfDNA quantity in plasma was significantly lower (Supplementary Figure 9). Before determining the best CpGs in the plasma cohort, we again tested the performance of CpGs that were selected in the test cohort. Breast cancer patients showed a non-significant higher methylation frequency for *SPAG6* (mean of 6.6% in benign controls versus 8.4% in breast cancer cases, *p* = 0.2536), *PER1* (2.6% versus 4.8%, *p* = 0.5792), *NKX2-6* (1.3% versus 2.9%, *p* = 0.1898) and *ITIH5* (6.8% versus 5.9%, *p* = 0.9343). Using these test cohort specific CpGs ROC curve analysis of single genes produced no significant results. A combination of *SPAG6* and *NKX2-6* revealed a sensitivity of 27% for breast cancer detection (cut-off methylation 6.9%, 85% specificity), which could be increased to 31% using a combination of *SPAG6* and *PER1* (cut-off 7.7%, 85% specificity). A combination of all four biomarker genes resulted in a sensitivity of 51% (cut-off 5.1%, 79% specificity). The highest sensitivity for breast cancer detection was however achieved with a combination of *SPAG6*, *PER1* and *NKX2-6* (58% sensitivity, cut-off 4.2%, 79% specificity). To uncover the most relevant CpGs in plasma, FC and GLM analysis were performed. The most discriminative CpGs in the plasma cohort were CpG1/2/4/9/10 for *SPAG6*, CpG1/2 for *PER1* and CpG1/4 for *NKX2-6* and *ITIH5*. *SPAG6* showed a significantly increased methylation frequency in breast cancer patients (4.4% versus 7.8%, *p* = 0.0059) whereas *PER1* (2.6% versus 4.8%, *p* = 0.5792), *NKX2-6* (4.6% versus 6.5%, *p* = 0.0570) and *ITIH5* (7.6% versus 6.1%, *p* = 0.1063) showed a non-significant increase. ROC curve analysis revealed that *SPAG6* alone detected breast cancer at 50% sensitivity (cut-off 7.1%, 85% specificity), which could be increased to 60% by adding *PER1* (cut-off 5.5%, 85% specificity). Sensitivity was slightly increased to 64% when adding *ITIH5* (cut-off 5.4%, 80% specificity). When using all four genes, the SNiPER panel, breast cancer was detected with a similar sensitivity of 63% at 80% specificity (cut-off 5.4%, Supplementary Table 3, Supplementary Figure 10).

## Discussion

Hypermethylation of promoter regions of genes is a frequent and early event in breast carcinogenesis, its detection in blood therefore shows promise as a non-invasive method for breast cancer detection. In the current study, we identified and evaluated *SPAG6*, *PER1* and *NKX2-6* as novel epigenetic biomarkers for liquid biopsy-based early breast cancer detection. The biomarkers were evaluated in two independent serum cohorts consisting of in total 251 breast cancer cases and 219 benign controls and a plasma cohort (*n* = 125). The high methylation frequency of these markers in early breast cancer tissue (pT1 tumors), as determined by TCGA analysis, suggested their potential for early breast cancer detection in blood cfDNA. *ITIH5* was included on basis of previous work [29], where we showed that a panel of *ITIH5* and *DKK3* could detect breast cancer with 41% sensitivity. In the present study, DCIS could be detected at 63% sensitivity and early invasive breast cancer at 51% sensitivity in the test cohort using *SPAG6* and *ITIH5*. Sensitivity for DCIS could be increased to 70% by adding *PER1* and *NKX2-6* to the panel. In the plasma cohort, on basis of *SPAG6*, *PER1* and *ITIH5*, sensitivity for breast cancer detection was 64%.

The promise of liquid biopsy-based hypermethylation biomarkers in breast cancer detection has been investigated in recent studies as well. Radpour et al. performed methylation analysis on a seven-gene panel and showed 91.7% coverage in serum and 92.6% coverage in plasma, with sensitivities ranging from 25 to 88% [24]. The six-gene panel used by Shan et al. was able to detect breast cancer at 78% sensitivity and 82% specificity in a serum cohort consisting of 749 samples [28]. Moreover, a study by Hoque et al. found a 62% sensitivity for breast cancer detection using a four-gene panel at 87% specificity [23]. Furthermore, Uehiro et al. were able to detect breast cancer at 86.2% sensitivity using a four-marker panel [26]. In addition, Salta et al. [25] reported breast cancer detection with 81.8% sensitivity using a three-gene panel. Despite similar sensitivities and specificities, the current study has some strengths compared to previous investigations. First, we used the publicly available breast cancer TCGA dataset including 1156 tissue samples for identification of potential biomarkers instead of a small set of samples or literature. This allowed analysis of a large number of potential markers that had not been previously investigated. Moreover, only pre-invasive DCIS (pTis) and early invasive breast cancer (pT1) were included in the serum cohorts which is, to the best of our knowledge, the largest serum cohort of very early breast cancer cases to date. Most studies included only a limited number of pTis or pT1 cancers, making it difficult to determine the value of biomarkers for the detection of these tumors. Furthermore, cfDNA methylation analysis was performed with pyrosequencing. A major advantage of pyrosequencing is the separate interrogation of CpGs. This single CpG resolution is of great importance as we showed that adjacent CpGs in the promoter region can be very heterogeneous in their methylation frequency and that the use of the most discriminative CpGs improves biomarker performance. qMSP, as used in our previous study and by others, does not provide single CpG resolution. In addition, we sought for an easy but accurate statistic to determine the most discriminative CpGs. We aimed to incorporate age in the statistics, as methylation levels tend to increase with age and suggest a model for identification of discriminative CpGs based on several methods. More precisely, to get a general idea of which CpGs show discriminative methylation a fold change can be performed. As a next step, the more stringent GLM with cofactor age should be performed. To get even more stringent, cases and controls should be matched one-to-one with a maximal age difference of 5 years. After matching of the samples, a paired *t*-test and ROC-analysis should be performed for every single CpG.

Despite promising results in the serum test cohort and plasma cohort, breast cancer detection proved challenging in the validation cohort. Analysis uncovered a significant inverse correlation between cfDNA concentration and methylation frequency, which hints to the importance of sample processing and type of analyte. Accurate sample processing is necessary to detect tumor-specific changes in serum or plasma and is probably the main reason for lack of sensitivity of (epi)genetic biomarkers [31, 32]. Most cfDNA originates from normal cells, only a minor fraction, possibly as small as 0.1%, is tumor derived [33, 34]. Cell lysis therefore needs to be avoided, to prevent release of large amounts of genomic DNA (gDNA), leading to false negative results [33, 35]. One of the important factors influencing the total amount of cfDNA is the time between blood draw and processing, delay can significantly increase the release of cfDNA from hematopoietic cells [36, 37]. For this purpose, blood collection tubes with stabilizing reagents, such as PAXgene, have been developed [33, 38]. Besides sample processing, the type of analyte e.g. serum or plasma, is another important pre-analytical consideration. Recent liquid biopsy studies suggest that plasma is, compared to serum, the better analyte [36, 39]. The total quantity of cfDNA is strongly elevated in serum compared to plasma [36, 40] and cfDNA isolated from serum shows a significant higher integrity than that of plasma [41], indicating the presence of contaminating gDNA. This is probably due to gDNA release by white blood cells during blood clotting which is necessary to obtain serum [39]. Regardless of differences in percentages of ctDNA, numerous reports describe an equally sensitive detection of *KRAS*, *TP53*, *BRAF* and *SMAD4* mutations in plasma and serum [42–44]. In addition, methylation can be sensitively detected in serum and plasma as reported by us and others [23, 24, 26, 29]. To address both pre-analytical issues, we collected whole blood in PAXgene tubes for plasma isolation and indeed, in plasma breast cancer could be detected with an increased sensitivity (64%) compared to serum. It should however be noted that the plasma cohort consisted of 49% pT1- and 51% higher stage (> pT1) breast tumors, which will probably have had a positive influence on breast cancer detection as larger breast tumors shed higher amounts of ctDNA into the bloodstream [17]. In addition, only a small cohort of plasma samples was analyzed and therefore further validation should be pursued.

An additional downside of the current study is that breast cancer specificity of the SNiPER panel was not fully tested. We did include controls with benign disease instead of healthy controls; benign disease is a potential source for hypermethylated cfDNA as well [45]. However, increases in *SPAG6*, *PER1*, *NKX2-6* and *ITIH5* promoter methylation may not be confined to breast cancer and therefore needs to be tested in non-breast cancer patients such as colorectal- and lung cancer patients, the second and third most common cancers in women [1]. Lastly, although pyrosequencing performed robust in our hands and made the identification of clinically relevant CpGs possible, the technical sensitivity of pyrosequencing with a limit of detection of 5–10% [46] is not optimal for methylation analysis in samples with small amounts of ctDNA [17]. The limits of detection for *SPAG6-*, *PER1-* and *NKX2-6* assays were below the amount of cfDNA used in the pyrosequencing reaction. Still, the percentage of actual ctDNA is much lower compared to cfDNA concentration. Targeted Next-Generation Sequencing (NGS), offering a single CpG resolution with a technical sensitivity of 1% [47], might provide a better alternative for ctDNA methylation analysis.

Although liquid biopsy remains challenging due to the importance of pre-analytics, the small amounts of ctDNA and the requirement of sensitive detection techniques we were able to identify *SPAG6*, *PER1* and *NKX2-6* as potential blood-borne biomarkers for early breast cancer detection, which showed in combination with *ITIH5* a sensitivity of 64% for breast cancer detection. Quantification of promoter methylation in ctDNA isolated from plasma might in the future be a sensitive and specific tool to complement current breast cancer detection strategies. Even though highly promising, further technical development and clinical validation of the SNiPER panel is required.

## Materials and methods

### Serum cohort

DCIS patient serum samples were provided by university medical center Schleswig-Holstein (UKSH, *n* = 31) and Patient’s Tumor Bank of Hope (PATH-Biobank, *n* = 26). Serum samples of women with early invasive breast cancer, i.e. tumor size < 2 cm (pT1), without lymph node involvement (pN0) and distant metastasis (pM0), were provided by the RWTH centralized biomaterial bank (RWTH cBMB, *n* = 37) and PATH-Biobank (*n* = 157). Age-matched serum samples of women with benign disease were provided by RWTH cBMB (*n* = 34) and university hospital Erlangen (*n* = 185). All patients gave informed consent for retention and analysis of their serum for research purposes (local ethical review boards of UKSH (ref. No. B327/10 and D470/14), university hospital Bonn for PATH-Biobank (ref. No. 255/06), university hospital RWTH Aachen (ref. No. EK-206/09) and university hospital Erlangen (ref. No. EK-3937)). Blood was drawn before starting any cancer-specific treatment or surgery. Blood samples from all study participants were obtained by venipuncture using the S-Monovette (Sarstedt, Nümbrecht, Germany). Samples were centrifuged at 1500 g for 10 minutes at room temperature and 1 ml serum aliquots were stored at –80°C or in liquid nitrogen until use. An overview of the clinical characteristics of the breast cancer patients is summarized in Supplementary Tables 4 and 5.

### Plasma cohort

Plasma samples of breast cancer patients (*n* = 111) and benign controls (*n* = 14) were obtained from Luisenhospital Aachen and UKSH. All patients gave informed consent for retention and analysis of their plasma for research purposes (university hospital RWTH Aachen (ref. No. EK-206/09) and local ethical review boards of UKSH (ref. No. B327/10 and D470/14)). Blood was drawn before starting any cancer-specific treatment or surgery. Blood samples from all study participants were obtained by venipuncture using PAXgene tubes (Qiagen, Hilden, Germany). Samples were centrifuged at 2500 g for 15 minutes at room temperature, and 1 ml plasma aliquots were stored at –80°C until use. An overview of the clinical characteristics of the breast cancer patients is summarized in Supplementary Table 6.

### Candidate gene selection

Infinium HumanMethylation450 BeadChip data from The Cancer Genome Atlas (TCGA) were analyzed for identification of biomarkers (normal *n* = 132, breast cancer *n* = 1024, Supplementary Table 7) [48]. Candidates were identified by comparison of five tissues of each subtype (healthy, luminal A, luminal B, basal-like and HER2-enriched) and selected on basis of three criteria: (1) absence of or low methylation frequency (< 10%) in normal breast tissue, (2) high methylation (> 50%) in primary breast tumor tissue and (3) high methylation level (> 40%) in basal-like breast cancer. We specifically selected genes with an increase in promoter methylation as this provides a gain of signal which is easier to detect than a loss of signal, especially in samples with a higher background signal [27]. In addition, we were interested in identifying biomarker candidates with a functional relevance in breast cancer: class II tumor suppressor genes are often silenced by promoter hypermethylation. Single CpGs in the promoter regions of the candidates were analyzed to select for regions with the highest differential methylation. A student’s *t*-test was performed to determine the significance of differences in methylation level between normal breast tissue and breast cancer. The selected candidate genes and CpGs within their promoters were then evaluated in the complete TCGA dataset as an initial validation.

### Candidate gene CpG methylation assay establishment

All pyrosequencing assays were designed using the PSQ assay design Software 1.0 (Qiagen), primer sequences are listed in Supplementary Table 8. To determine technical specificity of the assays a dilution series with increasing amounts of fragmented unmethylated- and decreasing amounts of fragmented *in vitro* methylated lymphocyte bisulfite DNA (100%, 75%, 50%, 25%, 12.5%, 5%, 1% and 0%) was used. In addition, spike-in experiments were performed using fragmented *in vitro* methylated lymphocyte DNA, to assess technical sensitivity. To this end, 5 ng, 2.5 ng, 1 ng, 0.5 ng, 0.1 ng, 0.01 ng and 0 ng of DNA were spiked into pooled serum or plasma, isolated using the QIAamp Circulating Nucleic Acids kit (Qiagen) and bisulfite converted.

### *In vitro* methylation

Hundred μg lymphocyte DNA was treated with CpG methyltransferase (M.SssI, NEB, Ipswich, England) in the presence of 32 mM S-adenosylmethionine, followed by purification with the QIAamp DNA Mini kit (Qiagen) according to manufacturer’s recommendations.

### DNA fragmentation

Unmethylated- and *in vitro* methylated lymphocyte DNA were fragmented (± 180 bp) by Adaptive Focused Acoustics technology (Covaris, Woburn, Massachusetts). Fragmentation was performed at the genomics facility of the interdisciplinary center for clinical research (IZKF), University hospital RWTH Aachen (http://www.chip-facility.rwth-aachen.de/).

### CfDNA isolation

CfDNA was extracted from 1 ml serum or plasma using the QIAamp Circulating Nucleic Acids kit (Qiagen) according to manufacturer’s protocol with slight modification: isolation was performed without the addition of carrier RNA [49] and cfDNA was eluted in 60 µl buffer AVE. CfDNA concentration was determined using the Qubit 2.0 and the Qubit dsDNA High Sensitivity Assay (Life Technologies, Wilmington, USA).

### Bisulfite conversion

Extracted serum or plasma cfDNA was bisulfite converted using the EZ DNA methylation kit (ZymoResearch, Orange, CA, USA) as described previously [50]. Bisulfite converted DNA was eluted in 22 µl Elution buffer.

### Pyrosequencing

To quantitatively assess methylation status of CpG dinucleotides in the promoter regions of the identified candidates, pyrosequencing was performed. Initial fragments, 110–140 bp in size, were amplified using the PyroMark PCR Kit (Qiagen). Methylation ratios for each CpG were subsequently quantified on the PyroMark96 ID device using the Pyromark Gold SQA reagents (Qiagen) as previously described [51]. Unmethylated and *in vitro* methylated lymphocyte DNA served as technical controls. Water blanks were included as negative controls.

### Statistics

Statistical analyses were performed with SPSS 25.0 (SPSS, Chicago, IL, USA) and GraphPad Prism 5.0 (GraphPad Software Inc., La Jolla, CA, USA). Different statistical strategies to define the most discriminative CpGs for each gene were evaluated: a) Fold change, dividing for each single CpG mean methylation frequency of breast cancer patients by the mean methylation level of controls. CpG dinucleotides with the highest FC were used for analysis. b) General Linear Model (GLM) statistics with cofactor age. c) Receiver-operating-characteristics (ROC) of single CpGs of each gene to determine significance, area under the curve (AUC), sensitivity, specificity and cut-off value. ROC was performed as well to evaluate the diagnostic performance of single biomarkers and biomarker combinations. Kruskal-Wallis with post hoc Dunn’s tests was implemented to test for differences in cfDNA concentration and methylation levels across sites. Spearman tests were used to determine correlations. The limits of detection for the different pyrosequencing assays were calculated on basis of the standard deviation of the residuals and the slope of the regression line. *P*-values below 0.05 were considered significant.

## SUPPLEMENTARY MATERIALS



## Abbreviations

BRAF: B-Raf proto-oncogene
CEA: Carcinoma embryonic antigen
cfDNA: Circulating free DNA
CpG: CpG dinucleotide
ctDNA: Circulating free tumor DNA
DCIS: Ductal carcinoma *in situ*
FC: Fold change
GLM: Generalized linear model
ITIH5: Inter-alpha-trypsin inhibitor heavy chain 5
KRAS: KRAS proto-oncogene
NKX2-6: NK2 homeobox 6
PER1: Period 1
ROC: Receiver Operating Curve
SMAD4: SMAD family member 4
TP53: Tumor protein p53
SPAG6: Sperm associated antigen 6
TCGA: The Cancer Genome Atlas
