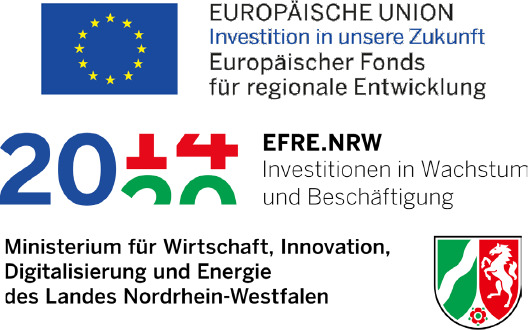# Correction: SNiPER: a novel hypermethylation biomarker panel for liquid biopsy based early breast cancer detection

**DOI:** 10.18632/oncotarget.27685

**Published:** 2020-07-28

**Authors:** Jolein Mijnes, Janina Tiedemann, Julian Eschenbruch, Janina Gasthaus, Sarah Bringezu, Dirk Bauerschlag, Nicolai Maass, Norbert Arnold, Jörg Weimer, Tobias Anzeneder, Peter A. Fasching, Matthias Rübner, Benjamin Bruno, Uwe Heindrichs, Jennifer Freres, Hanna Schulz, Ralf-Dieter Hilgers, Nadina Ortiz-Brüchle, Sonja von Serenyi, Ruth Knüchel, Vera Kloten, Edgar Dahl

**Affiliations:** ^1^ Molecular Oncology Group, Institute of Pathology, University Hospital RWTH Aachen, Aachen, Germany; ^2^ RWTH centralized Biomaterial Bank (RWTH cBMB) at the Institute of Pathology, University Hospital RWTH Aachen, Aachen, Germany; ^3^ Department of Gynecology and Obstetrics, University Medical Centre Schleswig-Holstein, Campus Kiel, Kiel, Germany; ^4^ Patients' Tumor Bank of Hope (PATH-Biobank) Foundation, München, Germany; ^5^ Department of Gynecology and Obstetrics, University Hospital Erlangen, Erlangen, Germany; ^6^ Institute of Clinical Molecular Biology, University Medical Centre Schleswig-Holstein, Campus Kiel, Christian-Albrechts-University, Kiel, Germany; ^7^ Department of Gynecology and Obstetrics Luisenhospital, Aachen, Germany; ^8^ Institute of Medical Statistics, University Hospital RWTH Aachen, Aachen, Germany; ^9^ Current address: Bayer AG, Pharmaceuticals Division, Biomarker Research, Wuppertal, Germany; ^*^ Share equal senior authorship

**This article has been corrected:** The Grant section information has been updated with the following logos:

Original article: Oncotarget. 2019; 10:6494–6508. 6494-6508. https://doi.org/10.18632/oncotarget.27303

**Figure:**