# Supplementary material for: SNiPER: a novel hypermethylation biomarker panel for liquid biopsy based early breast cancer detection
Source: Oncotarget. 2019 Nov 5;10(60):6494–508. doi: 10.18632/oncotarget.27303 (PMC6849652; doi:10.18632/oncotarget.27303)
Supplement: Supplementary file 1 [file oncotarget-10-6494-s001.pdf]

SNiPER: a novel hypermethylation biomarker panel for liquid biopsy based early breast cancer detection

SUPPLEMENTARY MATERIALS

Supplementary Table 1: Sensitivity and specificity of single markers and biomarker combinations in the serum validation cohort

		AUC	Significance	Sensitivity (%)	Specificity (%)	Cut-off (%)
PER1	invasive	0.5860	0.006157	20	82	3.8
	BC	0.5714	0.0179	19	82	
NKX2-6	DCIS	0.6587	0.0088	42	79	1.3
SPAG6 - NKX2-6	DCIS	0.6455	0.0164	38	80	4.3
PER1 - NKX2-6	DCIS	0.6337	0.0275	38	80	1.4
ITIH5 - NKX2-6	DCIS	0.6400	0.0209	50	77	2.9
SPAG6 - PER1 - NKX2-6	DCIS	0.6235	0.0416	31	83	3.5
SPAG6 - ITIH5 - NKX2-6	DCIS	0.6388	0.0221	35	81	4.4
SPAG6 - PER1 - ITIH5 - NKX2-6	DCIS	0.6229	0.0427	42	80	3.8

The following CpGs were used for ROC analysis: *SPAG6* CpG 2, 4 and 9, *PER1* CpG 1 and 2, *NKX2-6* CpG 3 and 4, *ITIH5* CpG 2 and 4. Only significant results are shown. AUC: Area under the curve, BC: breast cancer.

Supplementary Table 2: Sensitivity and specificity of single markers and biomarker combinations in the serum validation cohort

		AUC	Significance	Sensitivity (%)	Specificity (%)	Cut-off (%)
	DCIS	0.6755	0.0038	38	84	
***NKX2-6* CpG3**	Invasive	0.5823	0.0088	22	84	0.5
	BC	0.5955	0.0015	25	84	
	DCIS	0.5325	0.5914	15	79	
SPAG6 - PER1	Invasive	0.5849	0.0068	26	79	5.9
	BC	0.5774	0.0102	25	79	

The following CpGs were used for ROC analysis: *SPAG6* CpG 3, 4 and 8, *PER1* CpG 1 and 2, *NKX2-6* CpG 3, *ITIH5* CpG 2 and 3. Only significant results are shown. AUC: Area under the curve, BC: breast cancer.

Supplementary Table 3: Sensitivity and specificity of single markers and biomarker combinations in the plasma cohort, using the plasma cohort specific CpGs

	AUC	Significance	Sensitivity	Specificity	Cut-off (%)
			15%	100%	
SPAG6 **CpG1/2/4/9/10**	0.7265	0.005879	50%	85%	7.1
			56%	78%	
			23%	100%	
SPAG6 - PER1	0.7442	0.002983	60%	86%	5.5
			61%	71%	
			15%	100%	
SPAG6 - NKX2-6	0.7227	0.006778	42%	85%	7.2
			49%	79%	
			6%	100%	
SPAG6 - ITIH5	0.6853	0.0242	48%	85%	7.1
			58%	78%	
			23%	100%	
SPAG6 - PER1 - NKX2-6	0.7487	0.002493	46%	85%	6.2
			55%	78%	
			5%	100%	
SPAG6 - NKX2-6 - ITIH5	0.7024	0.01385	39%	85%	5.5
			51%	78%	
			7%	100%	
SPAG6 - PER1 - ITIH5	0.7056	0.01241	61%	86%	5.4
			64%	78%	
			6%	100%	
SPAG6 - PER1 - NKX2-6 - ITIH5	0.7288	0.005405	50%	85%	5.4
			64%	79%	

The following CpGs were used for ROC analysis: *SPAG6* CpG 1, 2, 4, 9 and 10, *PER1* CpG 1 and 2, *NKX2-6* CpG 1 and 4, *ITIH5* CpG 1 and 4. Only significant results are shown. AUC: Area under the curve, BC: breast cancer.

Supplementary Table 4: Clinicopathological parameters of invasive breast cancer patients in the test and validation cohort

	test cohort (n = 37)		validation cohort (n = 157)		
	N	%	N	%	p value[d]
**Age at diagnosis**					
median age	67		60		
< median	24	65	76	48	0.941
> median	13	35	78	50	
**Histological type**					
IDC	22	59	135	86	
ILC	10	27	18	11	0.001
Other	5	14	4	3	
Unknown	0	0	0	0	
**Tumour size[a]**					
pT1mic	0	0	1	1	
pT1a	3	8	4	3	
pT1b	13	35	39	25	0.102
pT1c	20	54	113	72	
Unknown	1	3	0	0	
**Lymph node status[a]**					
pN0	36	97	157	100	1.0
Unknown	1	3	0	0	
**Tumour grade[b]**					
G1	6	16	28	18	
G2	27	73	94	60	0.260
G3	4	11	35	22	
Unknown	0	0	0	0	
**ER[c]**					
Positive	21	57	136	87	
Negative	3	8	21	13	1.0
Unknown	13	35	0	0	
**PR[c]**					
Positive	18	49	123	78	
Negative	6	16	34	22	0.792
Unknown	13	35	0	0	
**HER2**					
Positive	0	0	15	10	
Negative	24	65	142	90	0.226
Unknown	14	38	0	0	

[a]TNM classification according to Sobin and Wittekind. [b]Grading according to Bloom and Richardson, as modified by Elston and Ellis. [c]ER: oestrogen receptor, PR: progesterone receptor, IRS: immunoreactive score according to Remmele and Stegner. IRS 0-2 was considered negative and IRS 3-12 was considered positive. [d]Two-sided Fischer Exact test. Percentages may not sum to 100% because of rounding.

Supplementary Table 5: Clinicopathological parameters of DCIS patients in the test and validation cohort

	test cohort (*n* = 31)		validation cohort (*n* = 26)		
	N	%	*N*	%	*p* value[d]
**Age at diagnosis**					
median age	67		61		
≤ median	17	55	12	46	0.153
> median	14	45	14	54	
**Histological type**					
DCIS	31	100	26	100	1.0
Unknown	0	0	0	0	
**Tumour size[a]**					
pTis	31	100	26	100	1.0
Unknown	0	0	0	0	
**Lymph node status[a]**					
pN0	1	3	14	54	< 0.001
Unknown	30	97	12	46	
**Tumour grade[b]**					
low grade	2	6	4	15	
intermediate grade	1	3	6	23	
non-high grade	2	6	5	19	0.850
high grade	3	10	11	42	
Unknown	23	74	0	0	
**ER[c]**					
Positive	15	48	19	73	
Negative	4	13	6	23	1.0
Unknown	12	39	1	4	
**PR[c]**					
Positive	14	45	18	69	
Negative	5	16	7	27	0.631
Unknown	12	39	1	4	
**HER2**					
Positive	0	0	3	12	
Negative	0	0	9	35	< 0.001
Unknown	31	100	14	54	

[a]TNM classification according to Sobin and Wittekind. [b] Grading according to Bloom and Richardson, as modified by Elston and Ellis. [c] ER: oestrogen receptor, PR: progesterone receptor, IRS: immunoreactive score according to Remmele and Stegner. IRS 0-2 was considered negative and IRS 3-12 was considered positive. [d] Two-sided Fischer Exact test. Percentages may not sum to 100% because of rounding.

Supplementary Table 6: Clinicopathological parameters of breast cancer patients in the plasma cohort

	N	%
**Age at diagnosis**		
median age	59	
≤ median	63	50
> median	62	50
Unknown	0	0
**Histological type**		
IDC	66	59
ILC	14	13
Other	4	4
Unknown	27	24
**Tumour size**[a]		
pT1	54	49
pT2	41	37
pT3	9	8
Unknown	7	6
**Lymph node status**		
pN0	64	55
pN1	24	22
pN2	6	5
pN3	2	2
Unknown	15	14
**Tumour grade**[b]		
G1	15	14
G2	54	49
G3	24	22
Unknown	18	16
**ER** [c]		
Positive (IRS 3–12)	90	81
Negative (IRS 0–2)	21	19
Unknown	0	0
**PR**[c]		
Positive (IRS 3–12)	70	63
Negative (IRS 0–2)	41	37
Unknown	0	0
**HER2**		
Positive	16	14
Negative	91	82
Unknown	4	4

[a]TNM classification according to Sobin and Wittekind. [b] Grading according to Bloom and Richardson, as modified by Elston and Ellis. [c] ER: oestrogen receptor, PR: progesterone receptor, IRS: immunoreactive score according to Remmele and Stegner. IRS 0-2 was considered negative and IRS 3-12 was considered positive.

Supplementary Table 7: Clinicopathological parameters of breast cancer patients in the TCGA cohort

	N	%
**Age at diagnosis**		
median age	58	
≤ median	607	53
> median	549	47
**Histological type**		
DCIS	0	0
IDC	842	73
ILC	195	17
Other	117	10
Unknown	2	0
**Tumor size**		
pT1c	291	25
pT2	677	59
pT3	141	12
pT4b	40	3
Unknown	7	1
**Lymph node status**		
pN0	535	46
pN1	392	34
pN2	127	11
pN3	76	7
Unknown	26	2
**ER**		
Positive (IRS 3-12)	560	48
Negative (IRS 0-2)	172	15
Unknown	424	37
**PR**		
Positive (IRS 3-12)	487	42
Negative (IRS 0-2)	242	21
Unknown	427	37
**HER2**		
Positive	103	9
Negative	619	54
Unknown	434	38
**Subtype**		
Luminal A	405	35
Luminal B	179	15
HER2 enriched	67	6
Basal-like	134	12
Normal-like	114	10

Percentages may not sum to 100% because of rounding.

Supplementary Table 8: Sequences of pyrosequencing primers

Gene	Primer	Primer sequence	Amplicon size
SPAG6	Forward primer	TAAGGAGTTTYGATTTTAGGGTAGTGTAGGGATATTT	114 bp
	Reverse primer	CCCRAACTCRAAAACTCAAACTACTAA	
	Sequencing primer	GGGTAGTGTAGGGATATT	
PER1	Forward primer	TTTAGTAGGGAAATGGGGGAGGG	103 bp
	Reverse primer	CCACRATCRCCAAAAAAACC	
	Sequencing primer	GAAATGGGGGAGGGG	
NKX2-6	Forward primer	GGGTGYGGAAGAGTTYGGAAAATTTTTAGTATTTG	123 bp
	Reverse primer	CAAAAAACTCCRAACCATCCAACTTTCT	
	Sequencing primer	GGAAAATTTTTAGTATTTG	
ITIH5	Forward primer	AGGGGTTYGTGGGGTTAATATAGGTGGTTT	138 bp
	Reverse primer	RCCRCTTCCCRACCTCAATCCC	
	Sequencing primer	GGGGTTAATATAGGTGGTT	

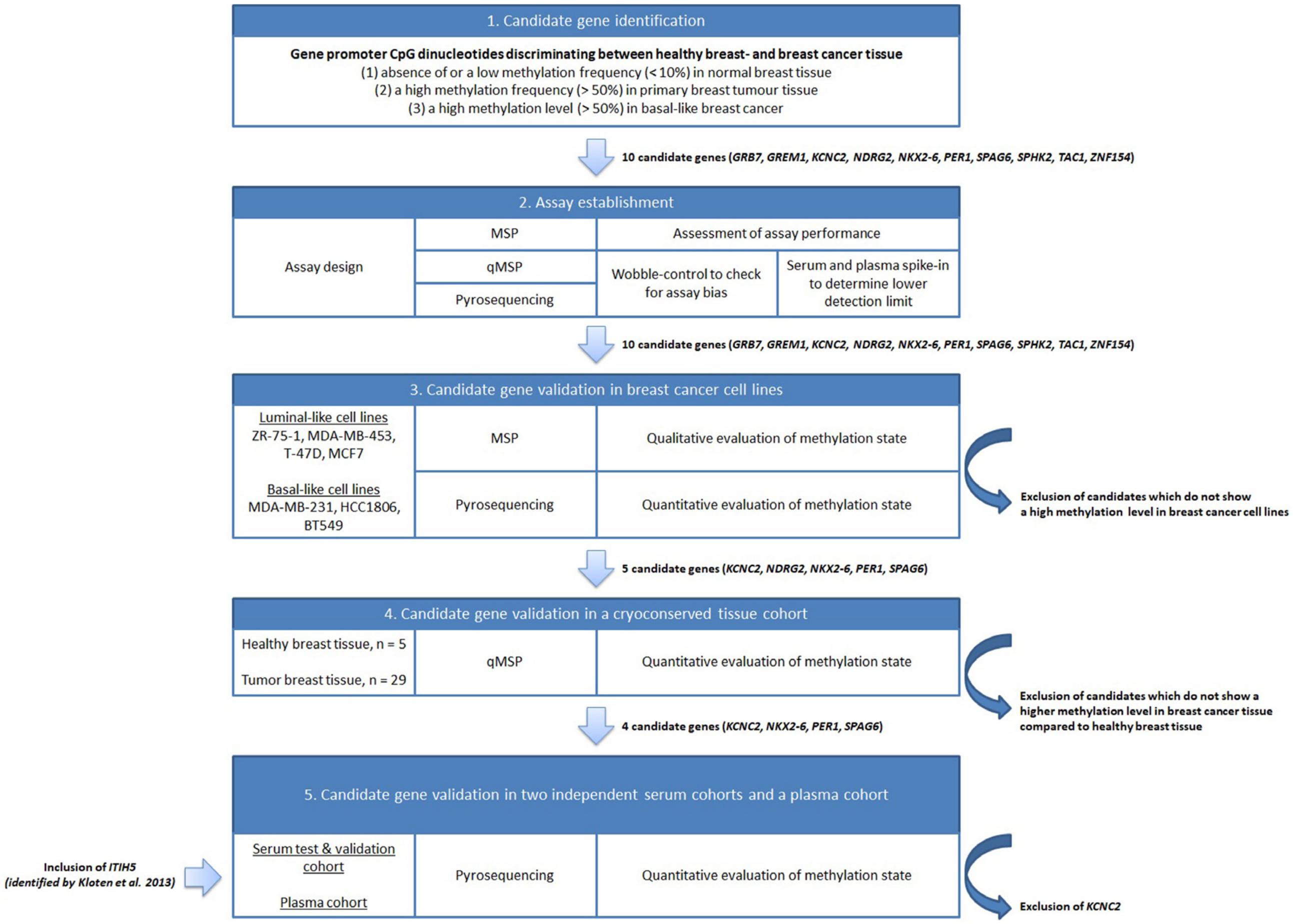

Supplementary Figure 1: Candidate gene validation and assay establishment. Candidate genes were identified using TCGA and assays for MSP, qMSP and pyrosequencing were designed. Assays were then evaluated for their specificity and sensitivity. As a first step in the validation of the candidates, methylation levels of all ten candidates was tested in luminal-like (MCF-7, MDA-MB-453, T-47D and ZR-75-1) and basal-like (BT-549, MDA-MB-231, HCC1806) breast cancer cell lines, reducing the potential biomarkers to only five. As a next step the remaining candidates were evaluated in a cohort of cryoconserved tissue of both healthy women (n = 5) and women with breast cancer (n = 29), which resulted in omission of a further candidate. The four remaining candidates, showing an increased methylation in breast cancer, were then tested in the serum test cohort. One of the four candidates showed not to be relevant and was not included in further analyses. *ITIH5* was included at this point, as it previously showed potential in blood-based breast cancer detection and the serum validation cohort and plasma cohort were analyzed.

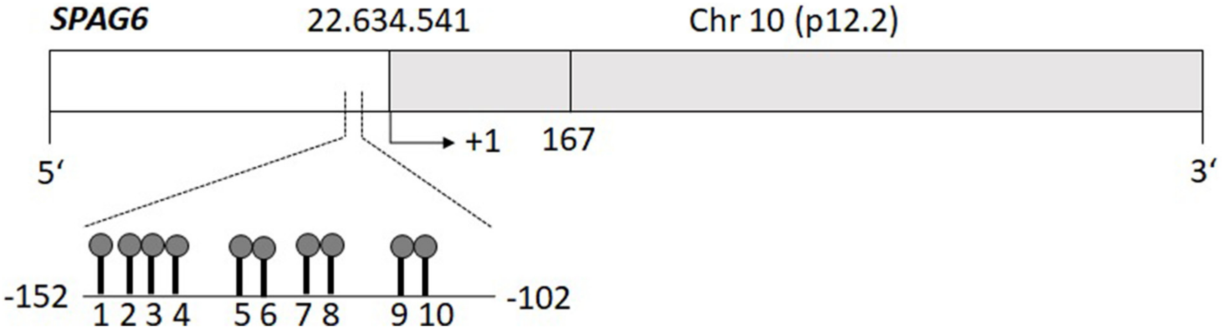

Supplementary Figure 2: Methylation map *SPAG6*. *SPAG6* is located on the short arm of chromosome 10. The pyrosequencing assay was placed before the transcription start site and included ten CpGs. CpG4 in the assay is cg18247055. Transcription start site (+1) and translation start site (arrow) are indicated.

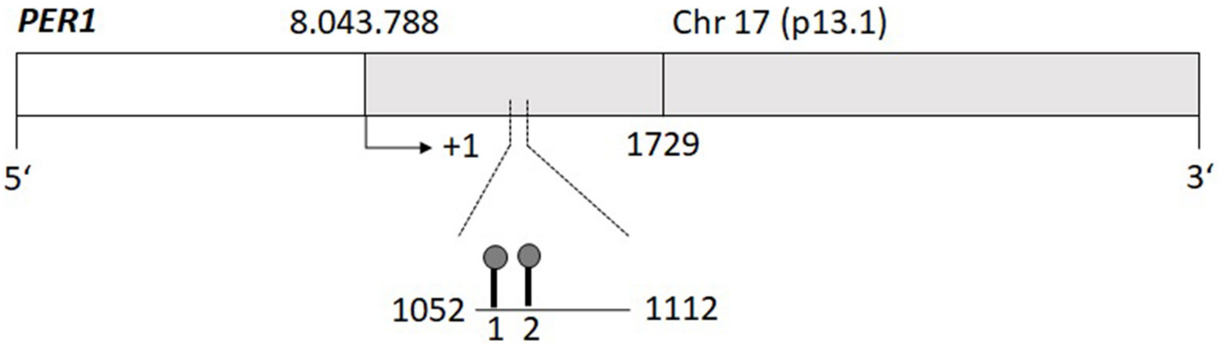

Supplementary Figure 3: Methylation map *PER1*. *PER1* is located on the short arm of chromosome 17. The pyrosequencing assay was placed in between the transcription start site and translation start site and included two CpGs. CpG2 in the assay is cg08521677. Transcription start site (+1) and translation start site (arrow) are indicated. Transcription start site (+1) and translation start site (arrow) are indicated.

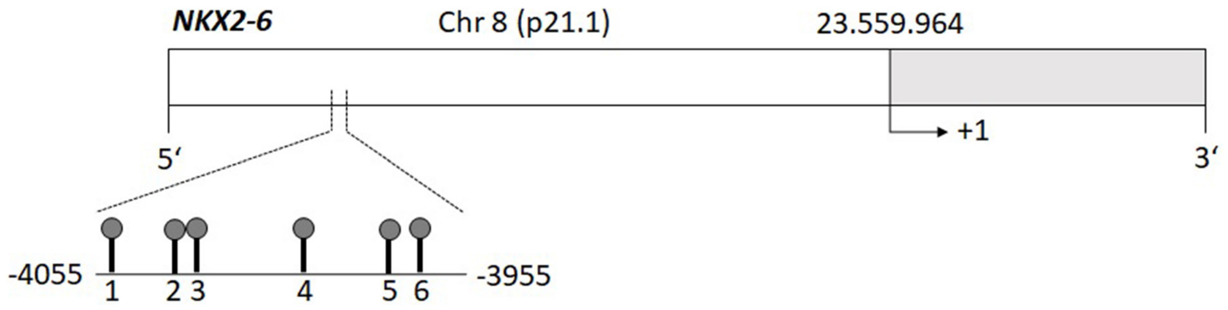

Supplementary Figure 4: Methylation map *NKX2-6*. *NKX2-6* is located on the short arm of chromosome 8. The pyrosequencing assay was placed before the transcription start site and included six CpGs. CpG6 in the assay is cg14428146. Transcription start site (+1) and translation start site (arrow) are indicated. Transcription start site (+1) and translation start site (arrow) are indicated.

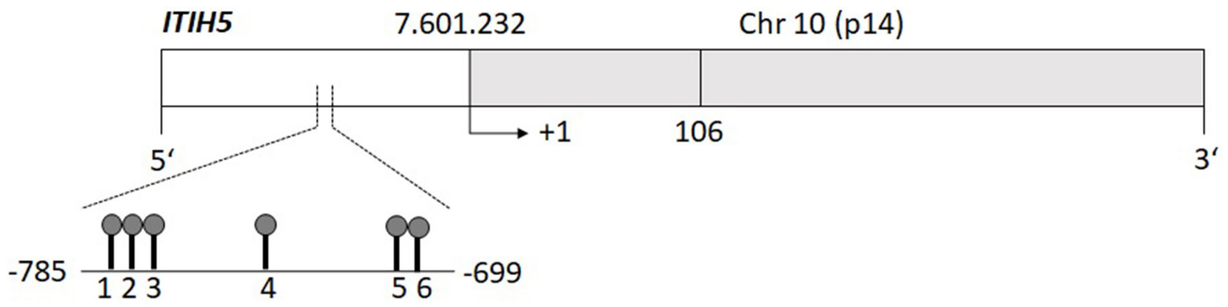

Supplementary Figure 5: Methylation map *ITIH5*. *ITIH5* is located on the short arm of chromosome 14. The pyrosequencing assay was placed before the transcription start site and included six CpGs. CpG1 in the assay is cg10119075. Transcription start site (+1) and translation start site (arrow) are indicated. Transcription start site (+1) and translation start site (arrow) are indicated.

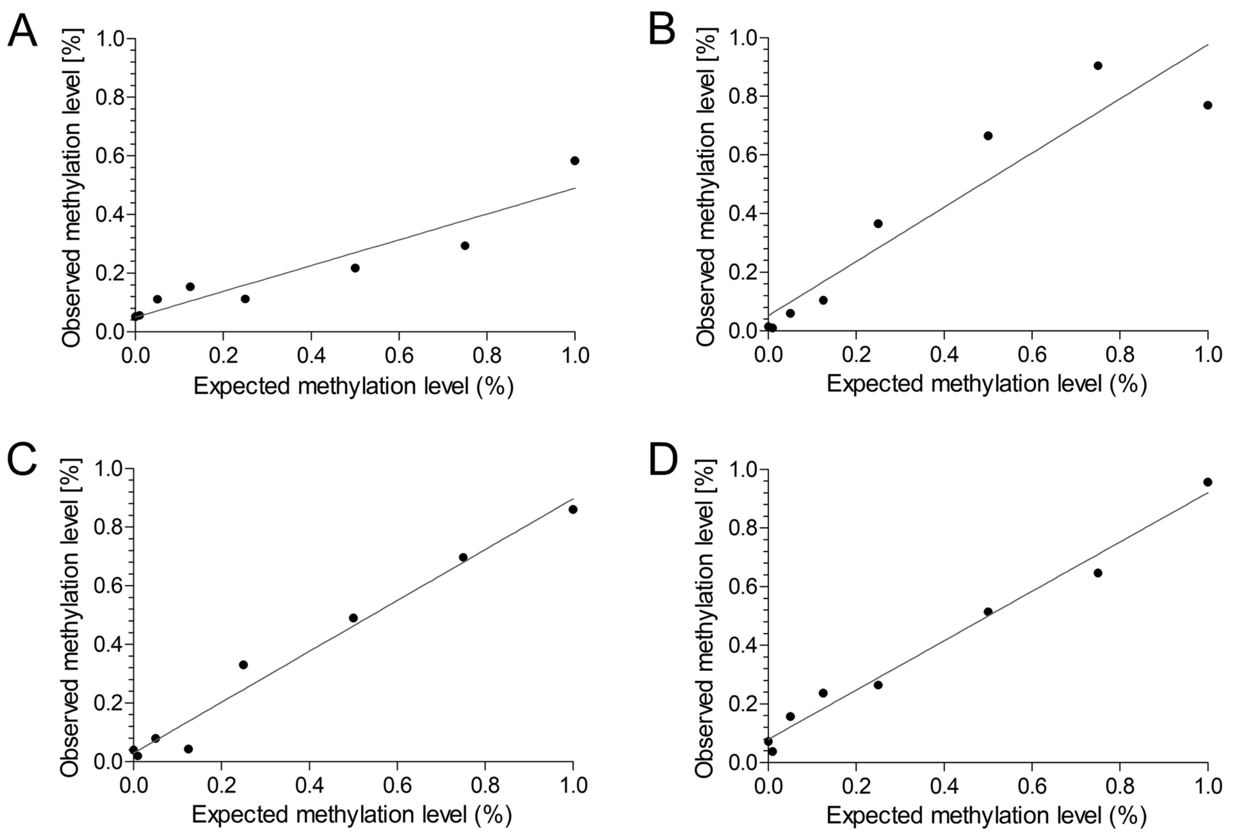

Supplementary Figure 6: Linear regression analysis shows high technical specificity of the used pyrosequencing assays. A dilution series with increasing methylation frequency was tested to determine a bias of the pyrosequencing assays towards either methylated or unmethylated sequences. *SPAG6* (**A**) and *PER1* (**B**) revealed a lower correlation coefficient (*SPAG6* R^2: 0.88 and *PER1* R^2: 0.88) than *NKX2-6* (**C**) and *ITIH5* (**D**) (*NKX2-6* R^2: 0.98 and *ITIH5* R^2: 0.98).

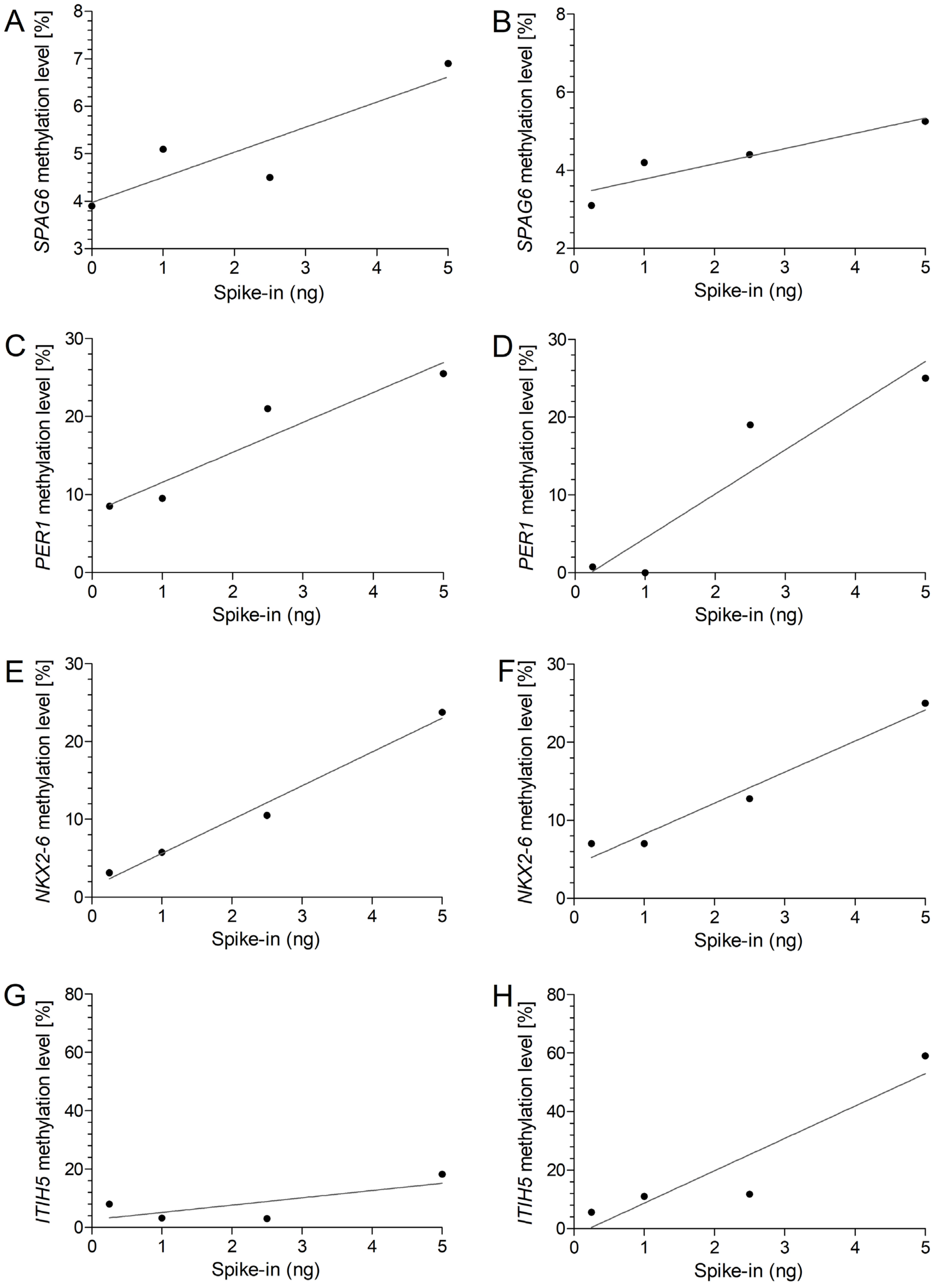

Supplementary Figure 7: Linear regression analysis shows high technical sensitivity, on basis of DNA spike-ins in serum and plasma. *In vitro* methylated fragmented DNA was spiked into pooled serum (from three donors) or pooled plasma (from four donors) and cfDNA was isolated according to standard protocol. Pyrosequencing was then performed to determine the lower detection limit of the assays. A linear regression analysis was performed on the obtained methylation values. Lines for *SPAG6* (**A** and **B**, serum R^2: 0.79, plasma R^2: 0.8593), *PER1* (**C** and **D**, serum R^2: 0.91, plasma R^2: 0.87), *NKX2-6* (**E** and **F**, serum R^2: 0.98, plasma R^2: 0.97) and *ITIH5* (**G** and **H**, serum R^2: 0.54, plasma R^2: 0.86) showed good correlations.

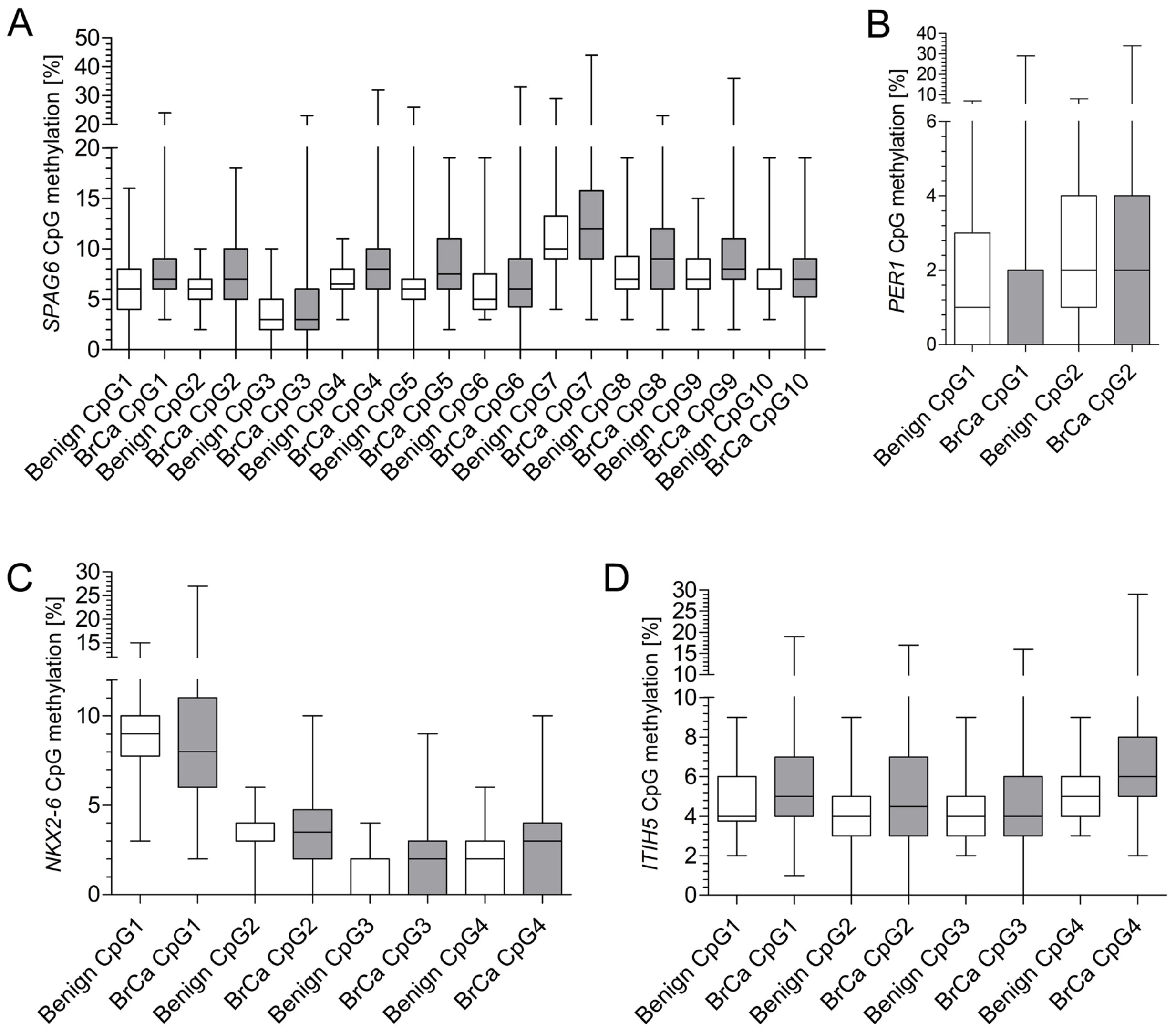

Supplementary Figure 8: Methylation frequency of candidate genes CpGs across a small promotor region shows a high level of heterogeneity in the test cohort. (**A**) *SPAG6* shows a variation in methylation of 4.2% to 13.2% in breast cancer cases and of 3.7% to 11.2% in benign cases. (**B**) Methylation levels for *PER1* vary between 2.9% and 3.9% in breast cancer cases and from 1.7% to 2.5% in benign controls. (**C**) methylation levels range from 2.0% to 9.0% in breast cancer cases for *NKX2-6*, whereas controls show a range of 1.5% to 8.9%. (**D**) For breast cancer patients *ITIH5* shows methylation levels between 4.9% and 6.5% and levels of 4.0% to 5.0% for controls. Boxes indicate 25% percentile, median and 75% percentile. Whiskers indicate minimum and maximum methylation levels. White bars: benign controls, grey bars: breast cancer cases. Whiskers indicate minimum, 25% percentile, median, 75% percentile and maximum.

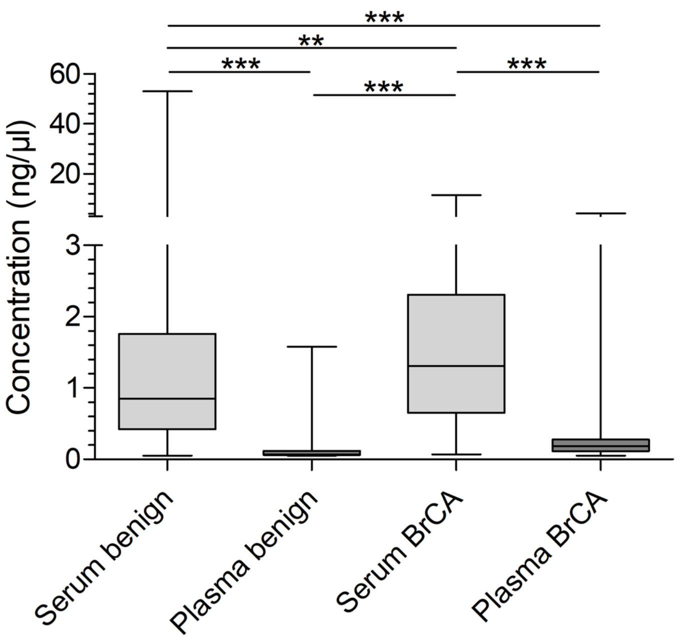

Supplementary Figure 9: cfDNA concentration in the serum and plasma cohort. cfDNA quantity in serum samples is significantly higher than in plasma samples. Benign and breast cancer serum samples show a significantly higher cfDNA concentration, compared to benign- and breast cancer plasma samples. $^{*}p < 0.05$, $^{**}p < 0.01$, $^{***}p < 0.001$, ns: non-significant. Whiskers indicate minimum, 25% percentile, median, 75% percentile and maximum.

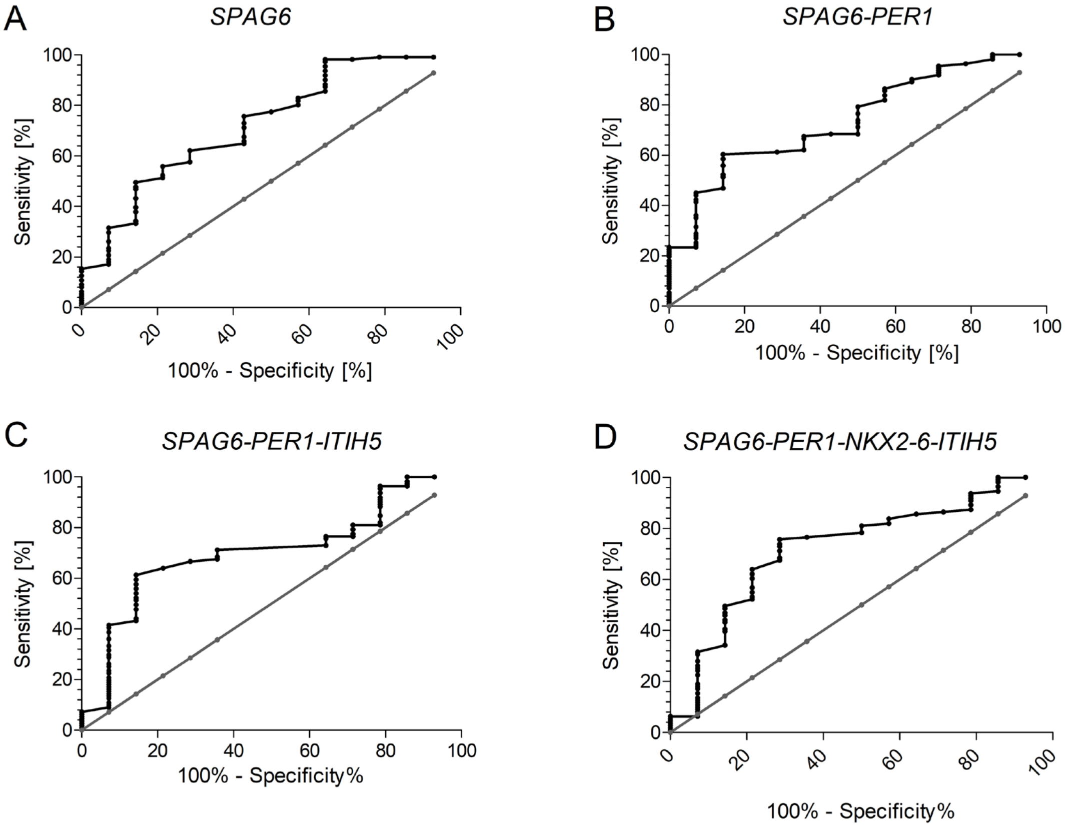

Supplementary Figure 10: ROC curve analysis for different biomarker panels in the plasma cohort reveals high sensitivity for breast cancer detection. (**A**) *SPAG6* alone already showed a 50% sensitivity for breast cancer detection (AUC: 0.7265, $p = 0.005879$), (**B**) which could be increased to 60% by the addition of *PER1* (AUC: 0.7442, $p = 0.002983$). (**C**) Sensitivity was slightly further increased to 64% when combining *SPAG6*, *PER1* and *ITIH5* (AUC: 0.7056, $p = 0.01241$). (**D**) An equal sensitivity was achieved when using all four biomarkers, the SNiPER panel, (AUC: 0.7288, $p = 0.005405$), with a 64% sensitivity for breast cancer detection. AUC: area under the curve, grey line: line of no discrimination.
